# 3D Biomechanics of Rugby Tackle Techniques to Inform Future Rugby Research Practice: a Systematic Review

**DOI:** 10.1186/s40798-021-00322-w

**Published:** 2021-06-07

**Authors:** Suzi Edwards, Roger Lee, Gordon Fuller, Matthew Buchanan, Timana Tahu, Ross Tucker, Andrew J. Gardner

**Affiliations:** 1 School of Environmental and Life Sciences, University of Newcastle, 10 Chittaway Rd, Ourimbah, NSW 2258 Australia; 2 Priority Research Centre for Stroke and Brain Injury, University of Newcastle, Callaghan, NSW Australia; 3 Priority Research Centre for Physical Activity and Nutrition, University of Newcastle, Callaghan, NSW Australia; 4 School of Health Science, University of Newcastle, Callaghan, NSW Australia; 5 Emergency Medicine Research in Sheffield Group, School of Health and Related Research, University of Sheffield, Sheffield, UK; 6 World Rugby, Pty (Ltd), Dublin, Ireland; 7 School of Medicine and Public Health, University of Newcastle, Callaghan, NSW Australia; 8 Hunter New England Local Health District Sports Concussion Program, Waratah, NSW Australia

**Keywords:** 3D motion capture, Tackle technique, Systematic review, Rugby league, Rugby union

## Abstract

**Background:**

The tackle is the most common in-play event in rugby union and rugby league (the rugby codes). It is also associated with the greatest propensity for injury and thus accounts for the most injuries in the sport. It is therefore of critical importance to accurately quantify how tackle technique alters injury risk using gold-standard methodology of three-dimensional motion (3D) capture.

**Objective:**

To examine the 3D motion capture methodology of rugby-style tackle techniques to provide recommendations to inform practice for future rugby code research and advance the knowledge of this field.

**Study Design:**

Systematic review.

**Methods:**

Articles published in English language, up to May 2020, were retrieved via nine online databases. All cross-sectional, correlational, observational, and cohort study designs using 3D motion capture of tackle techniques in rugby code players met inclusion criteria for this review. A qualitative synthesis using thematic analysis was pre-specified to identify five key themes.

**Results:**

Seven articles met eligibility criteria. Participant demographic information (theme one) involved a total of 92 rugby union players, ranging in skill level and playing experience. Experimental task design information (theme two) included one-on-one, front-on (*n*=5) or side-on (*n*=1) contact between a tackler and a ball carrier, or a tackler impacting a tackle bag or bump pad (*n*=3). 3D data collection (theme three) reported differing sampling frequencies and marker sets. 3D data reduction and analysis (theme four) procedures could be mostly replicated, but the definitions of temporal events, joint modelling and filtering varied between studies. Findings of the studies (theme five) showed that the one-on-one tackle technique can be altered (*n*=5) when tackle height, leg drive and/or tackle speed is modified. A study reported tackle coaching intervention.

**Conclusions:**

This is the first review to evaluate 3D motion capture of rugby-style tackle technique research. A research framework was identified: (i) participant demographic information, (ii) experimental task design information, (iii) 3D motion capture data specifications, and (iv) 3D data reduction and analysis. Adherence of future 3D tackling research to these framework principles will provide critical scientific evidence to better inform injury reduction and performance practices in the rugby codes.

**Trial Registration:**

The review was registered with PROSPERO (registration number CRD42018092312).

**Supplementary Information:**

The online version contains supplementary material available at 10.1186/s40798-021-00322-w.

## Key Points

- 3D motion capture is the gold standard methodology for analysing 3D tackle technique in the rugby codes. Research using this methodology is limited, and further research is required to understand the kinematics and kinetics of rugby-style tackle.
- The potential value of a tackling coaching intervention to alter tackle technique within a single session was highlighted by only one 3D biomechanics study.
- One-on-one tackle technique can be altered when tackle height, leg drive and/or tackle speed is modified, yet the most optimal tackle technique to guide coaches and clinicians to reduce the injury risk and/or optimise performance for both the ball carrier and tackler remains elusive.

## Introduction

Rugby league and rugby union (rugby codes) are popular, international, collision sports with 3.5 million registered rugby union players globally [1]. Game play in the rugby codes involves numerous physical collisions, where defensive players (known as tacklers) attempt to impede the progress of the attacking player (referred to as the ball carrier) to prevent the opposition from scoring. Depending on playing position, on average, a professional rugby league player will perform 11–30 tackles per player per game [2], whereas 4–14 successful tackles are performed by each player in elite level rugby union [3]. The top 50 tacklers in professional rugby league in 2020 performed between 33 and 54 tackles per game [4].

The tackle is the game play event in both rugby codes that is the most common mechanism of injury [5–7], leading to the greatest burden of injury for ankle (46%), knee (45%) and shoulder (66%) injuries [7] as well as serious head and spinal injuries [8]. Of concern is that the incidence, burden and severity of concussion [7]. Concussion is one of the most common injuries in rugby union (approximately 3.3–5.4 (95% CI 2.1 to 16.7) concussions per 1000 player match hours [9]) and rugby league (approximately 4.6 [5] to 8.0 (95% CI 4–18) concussions per 1000 player match hours [10]), along with contusions, muscle strains and ligament sprains [9, 10].

Two-dimensional (2D) video qualitative analysis studies have been used to extensively explore mechanisms for tackle-related injuries [11] and performance [12]. Substantial concerns have been raised with this subjective approach regarding (i) the quality and consistency of video analysis research in the rugby codes to understand performance [11, 12] and (ii) injury mechanism and/or risk factors [11] to inform the practice of coaches and clinicians [12]. Alternatively, quantitative 2D video analysis has been used to analyse a player’s in-game speed in rugby [13, 14], but this method is prone to inherent perspective error [14]. Perspective error refers to motion that is recorded outside of the plane of measurement [15]. This error also reduces the reliability of quantitative 2D video analysis to calculate joint angles during movement. To overcome perspective error, as well as other issues affecting the reliability and validity of 2D video analysis in sporting movements such as low sampling rate, camera resolution and sufficient lighting [16], three-dimensional (3D) motion capture methodology (i.e. optoelectronic) may be utilised [15]. Given that the tackle is a complex multi-planar movement, optoelectronic 3D motion capture analysis is considered the gold standard for 3D quantitative motion analysis, as it has the capacity to accurately quantify key kinematic tackling variables within an indoor laboratory and/or outdoor training environment. No reliable or valid 3D motion analysis technology currently exists that can be used to retrospectively analyse in-game 3D tackle biomechanics. Although considered the gold standard technique, the reliability of 3D tackle biomechanics is known to be adversely affected by the data collection methods, and reduction and analysis procedures [17, 18]. If tackle research using 3D motion capture is to inform the improvement of tertiary injury reduction programme, it is paramount that high-quality biomechanical research is undertaken, ensuring an appropriate research framework. Four key influences on the repeatability of the experimental design and quality include participant demographic information, experimental task design, 3D motion capture data collection and reduction and analysis procedures.

In view of the importance of the tackle for a team’s performance, the number of tackle events that occur during match play in both rugby codes and the risk for injury to the tackler and ball carrier, this review aimed to examine the optoelectronic 3D motion capture methodology of rugby-style tackle techniques to provide recommendations to inform practice for future rugby-code research and advance the knowledge of this field.

## Methods

This review adhered to the Preferred Reporting Items for Systematic Reviews and Meta-Analyses (PRISMA) statement [19]. The review was registered with PROSPERO (registration number CRD42018092312).

### Search Strategy

**Fig. 1:**
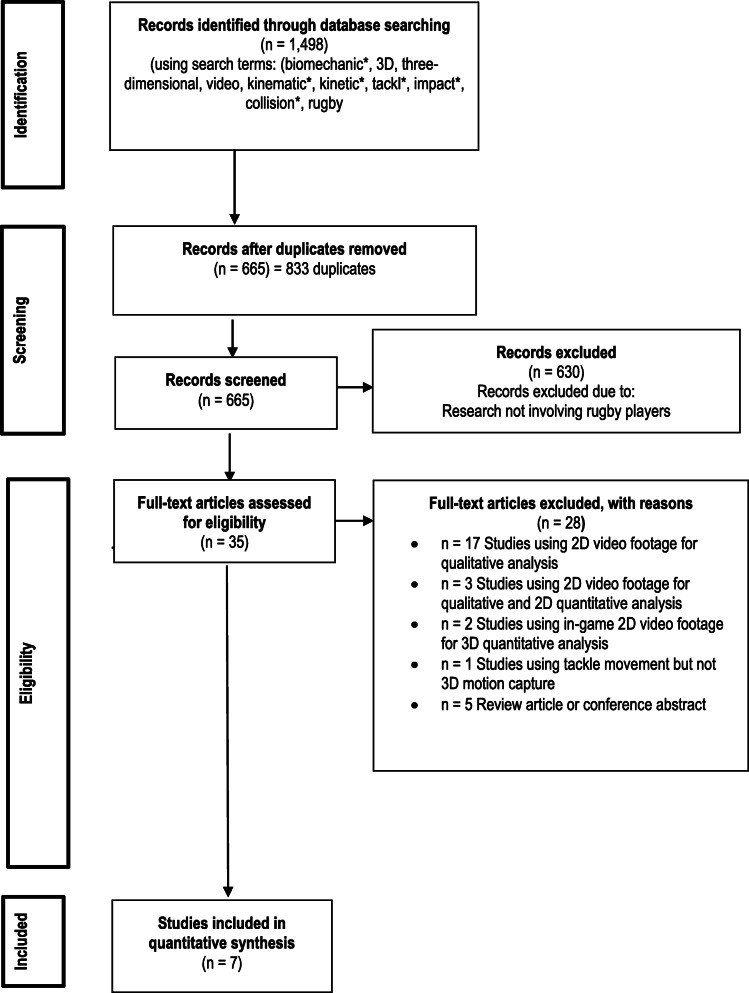
PRISMA diagram adhered to for including articles in this review

The literature search was conducted using nine databases: MEDLINE, SPORTDiscus, Web of Science, Scopus, CINAHL, Proquest Research Library, PubMed, Embase and Cochrane Library, for eligible articles (Additional file 1: Appendix 1). Articles were limited to those that were peer reviewed and published in English-language journals up to 13 May 2020. The search strategy used was (biomechanic* OR 3D OR three-dimensional OR video OR kinematic* OR kinetic*) AND (tackl* OR impact* OR collision*) AND (rugby). The reference lists of each study meeting inclusion were also reviewed for further potentially eligible studies. The result of the search strategy and eligibility review is presented in the PRISMA flow diagram presented in Fig. 1.

### Eligibility Criteria

All studies involving a cohort, observational, cross-sectional or correlational design, and including active, adult, rugby union or rugby league players using 3D motion capture were considered to meet eligibility criteria for inclusion in this review. There was no restriction placed on the level of competition. Studies were excluded if they were conference abstracts, systematic reviews, thesis dissertations, editorials, expert opinions, letters to the editor or case studies. Studies were also excluded if they (i) examined non-tackle, rugby-related movements (e.g. change of direction task or goal kicking), (ii) used only a 2D analysis or (iii) used only an inertial measurement unit (accelerometers, magnetometers and/or gyroscopes). Non-English language publications, studies involving participants of sports other than rugby league or rugby union, studies involving children or adolescent athletes and studies including 3D equipment other than motion capture were also excluded from the review.

All citations identified within the initial search were imported into Covidence (Veritas Health Innovation Ltd, Victoria, Australia). Two authors (MB, RL) independently screened the title and abstract of all citations to determine whether the article met the eligibility criteria. If there was uncertainty about whether a study should be included based on the review of the title and abstract, the full article was retrieved. Both authors independently completed full-text reviews of all retrieved articles. In the event of conflict regarding eligibility following the full-text review, a consensus discussion with the third author (SE) was conducted to resolve discrepancies.

### Risk of Bias Assessment

Two authors (MB and RL) used the Downs and Black checklist [20] to examine the risk of bias of all eligible articles. This checklist has been reported to possess good test-retest and inter-rater reliability for assessing study quality [20]. Any inconsistencies between the two raters on any of the Downs and Black checklist item scores were resolved by a third author (AG), who independently scored those items. This was the final arbitering score. The classification of methodological risk of bias was considered as follows: low ≥75%, moderate 61–74% and high ≤60%.

### Data Extraction and Qualitative Synthesis

The data extracted from the eligible studies included the following five key areas: (i) *theme one—participant demographic information*: participant inclusion/exclusion criteria, rugby code, skill level, sample size, sex, age, mass, height, playing experience, playing position(s). (ii) *Theme two—experimental task design information*: type of tackle, total number of tackles completed within the session, tackle instruction to ball carrier and/or tackler, tackle intensity, the side of shoulder engagement of tackler, rest period between trials, whether the ball carrier was taken to ground, surface type, rest between trials, experimental task conditions if applicable. (iii) *Theme three—3D motion capture data specifications*: camera type, sampling rate, marker set, data collection volume. (iv) *Theme four—3D data reduction and analysis*: filtering method, 3D modelling information, definition of temporal event(s) during the tackle, speed of the ball carrier and/or tackler prior to and at contact, segment angle, joint angle, range of motion, peak linear and angular acceleration and velocity. (v) *Theme Five—research findings*: significant findings reported by the eligible studies.

All extracted data were collected from the reported variables that occurred during phases of the tackle, at the point of contact, or throughout the tackle movement. Angle variables were reported as degrees (°); accelerations were reported as either m·s^2^, rad·s^−2^, or G (acceleration due to gravity); and other variables measured were reported as either percentage (%), kilonewtons (kN), seconds (s), metres per second (m/s) or body weight (BW). When extracting the data, the summary measures of the means and standard deviations were reported. Corresponding authors were contacted to provide mean and standard deviations of variables that were in graphical form or when such data could not be located within the publication.

A qualitative synthesis using thematic analysis was pre-specified in this study to identify key themes [21] of the limited rugby-code literature on the 3D kinematics of the tackler.

## Results

### Search of the Literature

A total of 1498 articles were identified through the initial search strategy (see Fig. 1), from which 833 duplicates were identified and removed. The titles and abstract of 665 articles were then screened with 630 articles subsequently excluded. The full texts of 35 articles were retrieved and reviewed: 17 articles were identified as using 2D video footage for qualitative analysis; three studies used 2D video footage for qualitative and 2D for quantitative analysis; two articles used in-game 2D video footage for 3D quantitative analysis; one study used 3D equipment other than motion capture; and five articles were either conference abstracts or review articles. Seven articles met inclusion criteria.

### Risk of Bias

The scores of each Downs and Black checklist item and the overall quality index for all the included studies are presented in Table 1. Agreement was achieved on 164 (87%) of the Downs and Black checklist items by the two raters. For all 25 scores that were not agreed upon by the two raters, the risk of bias scores from the third reviewer were used to reach a consensus. One study was categorised as moderate risk of bias [23] and six studies as high risk of bias. Three studies achieved a methodological score of 15 [22, 25, 26], three studies achieved a score of 16 [24, 27, 28], and one study scored 20 [23].

**Table 1:** Downs and Black risk of bias scores

Downs and Black checklist item	Tierney et al. [22]	Tierney and Simms [23]	Wundersitz et al. [24]	Kerr et al. [25]	Seminati et al. [26]	Tanabe et al. [27]	Kawasaki et al. [28]
1. Study aim/hypothesis clearly defined	1	1	1	1	1	1	1
2. Clear definition of main outcomes	1	1	1	1	1	1	1
3. Patient characteristics clearly defined	1	1	1	1	1	1	1
4. Interventions used are clearly defined	1	1	1	1	1	1	1
5. Clearly defined confounders in subject groups	2	2	2	2	2	2	2
6. Study main findings are clearly defined	1	1	1	1	1	1	1
7. Random variability estimates given for main outcome data	0	1	0	0	0	0	0
8. Clearly defined adverse events that may have influenced intervention	0	0	0	0	0	0	0
9. Patients not followed up have characteristics clearly defined	0	0	0	0	0	0	0
10. Main outcome(s) probability values clearly reported	1	1	1	1	0	1	1
11. Subjects asked to participate were representative of source population	0	0	0	0	0	0	0
12. Subjects prepared to participate were representative of source population	0	0	0	0	0	0	0
13. Location and delivery of study treatment were representative of source population	0	0	0	0	0	0	0
14. Opportunity taken to blind test subjects from intervention	0	0	0	0	0	0	0
15. Opportunity taken to blind researchers from intervention	0	0	0	0	0	0	0
16. Clearly defined results subject to ‘data dredging’	0	1	0	0	0	0	0
17. Analysis adjusted for different lengths of follow-up	0	0	0	0	0	0	0
18. Use of suitable statistical tests for main outcome assessment	1	1	1	1	1	1	1
19. Reliable intervention adherence	1	1	1	1	1	1	1
20. Measures for main outcomes reliable and valid	1	1	1	1	1	1	1
21. All participants recruited from the same source population	0	1	0	0	0	0	0
22. Similar period of time used to recruit test subjects in different intervention groups	0	1	0	0	0	0	0
23. Random methods used to assign test subjects	0	0	0	0	0	0	0
24. Patients and researchers blinded to intervention until completion of recruitment	0	0	0	0	0	0	0
25. Analysis of main findings allow for sufficient confounding adjustment	0	0	0	0	0	0	0
26. Lost to follow up patients addressed	0	0	0	0	0	0	0
27. Power sufficient for study	4	5	5	4	5	5	5
**Quality index score (out of 31)**	15	20	16	15	15	16	16
**Quality percentage index (%)**	48	65	52	48	48	52	52

### Theme One—Participant Demographic Information

The characteristics of the seven eligible studies are presented in Table 2. There were a total of 92 rugby union participants (individual study numbers ranged from 4 [22, 23] to 25 [24]) across the seven eligible studies. Level of play included high school [25], community [26], collegiate [25–27], semi-elite [24] and professional [22, 23, 27, 28]. Three studies used mixed cohorts [25–27], one study included both male and female participants and reported sex results separately [25], one study included male players only [24], and three studies did not report the sex of the participants [22, 23, 28]. Playing experience, when reported, ranged from less than 6 months [25] to an average of over 11 years [27]. Playing position was only reported in three (43%) of eligible articles [25, 27, 28], where 22 backs and 16 forwards were included in those studies.

**Table 2:** Theme 1—participant demographic information

Reference	Sport	Skill level	Sample size	Age (years)	Body mass (kg)	Height (cm)	Playing experience (years)	Positions
Tierney et al. [22]	Rugby union	Professional	4 M	—	—	—	—	—
Tierney and Simms [23]	Rugby union	Professional	4 M	—	—	—	—	—
Wundersitz et al. [24]	Rugby union	Semi-elite	25 M	23.3 ±4.3	96.5 ± 18.1	180 ± 6	—	—
Kerr et al. [25]	Rugby union	College	10 (8 M, 2 F)	21.4	M 89, F 72.1	M 180.0, F 168.9	Novice <1 year, experienced >1 year. All players, 1–4 years	6 backs, 4 forwards
		College	20 (8 M:12 F)	21.1	M 90.2, F 66.8	M 174.0, F 166.8	All players 0.5–2.8 years at college; number of years, novice 0.1–0.5 months; experienced mean 2 years 8 months	—
		High school	20 (12 M: 8 F)	16.0	74.3	—	0.2–4.0 years high school Mean 12 months	—
Seminati et al. [26]	Rugby union	Community, college	15 M	23.5 ± 5.1	96.6 ± 12.9	182 ± 6	Minimum of 3 years playing experience at community or university level	—
Tanabe et al. [27]	Rugby union	College, professional	15 M (11 college, 4 professional)	22.3 ± 1.9 (21.2 to 23.2)	83.9 ± 13.6 (76.3 to 91.4)	175.3 ± 6.9 (171.5 to 179.1)	11.7 ± 3.6 (9.7 to 13.7)	9 backs, 6 forwards
Kawasaki et al. [28]	Rugby union	College, professional	13 M (9 college, 4 professional)	23.2 ± 3.8 (21.3 to 25.0)	83.7 ± 12.9 (77.5 to 89.9)	175.6 ± 6.5 (172.4 to 178.7)	10.6 ± 3.9 (8.7 to 12.5)	7 backs, 6 forwards

Note. *M* male, *F* female

### Theme Two—Experimental Task Design Information

Experimental task characteristics varied widely in the eligible articles (Table 3). One-on-one, front-on [22–25, 27] or side-on [27] contact between a tackler and a ball carrier was used in five studies [22–25, 27], while three studies used a tackler impacting a tackle bag or bump pad [24, 26, 28]. Only half the articles reported detailed tackle task description on the intensity in which to engage in the tackle [24–28], whether the tackle was completed to ground [24–26], if they employed a dominant side shoulder engagement in the tackle [25–28], the experimental surface type [24, 25], the approach distance [22–24, 26, 27] or the rest interval between trials [24–26].

**Table 3:** Experimental task design (theme 2), 3D motion capture data collection (theme 3) and 3D reduction and analysis procedures (theme 4)

Variable		Kerr et al. [25]	Tierney et al. [22]	Tierney and Simms [23]	Seminati et al. [26]	Kawasaki et al. [28]	Tanabe et al. [27]	Wundersitz et al. [24]
**Experimental task**	**Task**	Front-on tackle	Front-on tackle	Front-on tackle	Tackle bag	Tackle-bag	Side-on tackle Front-on tackle	Front-on tackle, tackle bag, bump pad
	**Intensity**	Training	—	—	Match play	Match play	Match play	Match play
	**Tackle to ground**	Yes	—	—	No	—	—	Yes
	**Approach distance**	—	2.5 m	2.5 m	3 steps	—	10 m	5 m, 6 m, 10 m
	**Shoulder side engagement**	Dominant (unknown)	—	—	Dominant (right)	Dominant (right)	Dominant (right)	
	**Surface**	6 cm polyethylene, polyethylene foam	—	—	—	—	—	Outdoor rugby field
	**Approach speed**	Yes	Yes	Yes	No	No	No	No
	**Trials**	Phase 1: 5 trials per 10 participants; phase 2: 3 trials pre- and post-intervention per 20 participants	5 trials per 4 participants	5 trials per 4 participants	Up to 20 trials per 15 participants	66 successful trials amongst 13 participants	65 successful trials amongst 15 participants	10 bump pad trials (*n*=250), 10 tackle bag trials (*n*=250) and 5 front-on tackle trials (*n*=125) per 25 participants
	**Rest intervals**	Phase 1: 2-min; phase 2: unknown	—	—	>1-minute	—	—	1-min trials 5-min between tasks
**Data collection**	**Motion capture system**	12-camera MX3 Pro, Vicon	10-camera Bonita-B10, Vicon and 3-camera Bonita 720C, Vicon	10-camera Bonita-B10, Vicon and 3-camera Bonita 720C, Vicon	16 cameras Oqus, Qualysis	20-camera MX, Vicon	20-camera MX, Vicon	12-cameras Raptor E, Motion Analysis Corp
	**Sample rate**	—	200 Hz, 67 Hz	200 Hz, 67 Hz	250 Hz	250 Hz	250 Hz	500 Hz
	**Data collection volume**	5 m × 5 m	—	—	—	5 x 10 m	10 x 10 m	—
	**Markers**	40 (body suit)	43 (plug-in-gait)	43 (plug-in-gait)	64 (custom)	38 (custom)	38 (custom)	1 on wearable sensor
**Data reduction and Analysis**	**Filtering**	None, based on previously established technique	Zero-lag, fourth-order *f*_c_=15 Hz (*110 Hz)	Zero-lag, fourth-order *f*_c_=15 Hz (*110 Hz)	Low pass, zero-lag, fourth-order Butterworth, *f*_c_=16 Hz	Low pass, zero-lag, fourth-order Butterworth, *f*_c_=6 Hz	Low pass, zero-lag, fourth-order Butterworth, *f*_c_=6 Hz	Low pass, zero-lag, fourth-order Butterworth, *f*_c_=10Hz
	**Joint coordinate system**	No	Yes (plug-in-gait)	Yes (plug-in-gait)	No	Yes (ISB)	Yes (ISB)	—
	**3D rotations defined**	No	Yes	Yes	No	Yes, “typical” Euler sequence	Yes, “typical” Euler sequence	—
	**Joint angle defined**	Yes, 2D angle	—	—	—	Yes	Yes	—
	**Head linear and angular kinematics defined**	Yes	Yes	Yes	—	—	—	—
	**Temporal event definition**	Yes	No	No	Yes	Yes	Yes	Yes

### Theme Three—3D Data Collection

Every eligible study captured 3D tackling data with a passive optoelectronic 3D motion analysis system with various sampling frequencies ranging from unknown [25] up to 500 Hz [24] in an indoor laboratory [22, 23, 25–28] or in an outdoor training environment on a rugby field [24]. Whole body retroreflective marker sets ranged from a 40 marker suit [25] to 64 customised marker set [26] (Table 3).

### Theme Four—3D Data Reduction and Analysis

All but one study [25], employed a low pass zero-lag fourth order filtering with differing cutoff frequencies. Most studies did provide sufficient information to replicate their 3D model used to calculate kinematic variables such that four studies reported their joint co-ordinate system definition [22, 23, 27, 28], yet only two studies defined their 3D rotation [27, 28].

Definitions of the time of temporal events varied between studies. The moment of impact in a one-on-one, front-on tackle was defined as a sudden decrease in the tacklers and ball carriers velocity [25], initial displacement of the ball carrier’s markers [27] or was not defined [22, 23]. Impact in a tackle bag task was defined as the instant when the tackle bag horizontal velocity of the centre of the mass reached its highest velocity [26] or the initial displacement of the marker on the tackle bag [28].

### Theme Five—Findings of Studies

Joint angles were reported in the four eligible studies [26–28] and are presented in Table 4. Studies reported a variety of joint angles including the neck [26–28], trunk [26–28], hip [25, 28], knee [25, 28], shoulder [27, 28] and ankle [28]. Other variables of interest included in the eligible studies such as head acceleration [22, 23, 25] or player speed [22, 23] are reported in Table 5.

**Table 4:** Theme 5 - Findings of the studies: 3D joint angle of the tackler during a tackle task.

	Kinematic variable						Significant findings
Kerr et al. [25]	Contact		*Sample of collegiate players*				Descriptive data only
		Knee flexion (°)	Right: 108.0 ± 28.8	Left: 122.5 ± 25.8			
		Hip flexion (°)	Right: 97.7 ± 21.5	Left: 98.5 ± 21.7			
		Cervical spine (°)	147.0 ± 25.8				
	ROM	Knee flexion (°)	82.0–141.0				
		Hip flexion (°)	81.0–120.7				
		Cervical spine (°)	129.7–163.5				
	Contact	Knee flexion (°)	*Skilled (male + female)*	*Novice (male + female)*	*Skilled (male + female)*	*Novice (male + female)*	↑ knee flexion after video intervention versus before in skilled male and female players (*P* = 0.02), and female skilled players (*P* < 0.01) ↑ right knee flexion in skilled male and female players post-intervention (*P* = 0.04), male players pre-intervention (*P* = 0.01), and female players post-intervention (*P* = 0.01).
			Pre-intervention	Pre-intervention	Post-intervention	Post-intervention	
			77.2 ± 28.9	64.3 ± 7.9	117.6 ± 35.8	79.3 ± 28.3	
		Hip flexion (°)	73.1 ± 22.0	65.5 ± 14.3	68.5 ± 21.7	73.6 ± 15.2	NS
Seminati et al. [26]	Contact		*Dominant shoulder stationary*	*Non-dominant shoulder stationary*	*Dominant shoulder In-motion*		
		Neck flexion (°)	22 ± 15	27 ± 19	27 ± 15		↓ neck flexion in dominant shoulder side in a stationary position than non-dominant shoulder side in a stationary position (d±90% CI = −0.26± 0.36) or dominant shoulder side when in-motion (d±90% CI = −0.34 ± 0.21)
		Neck lateral flexion (°)	12 ± 9	18 ± 10	12 ± 8		↓ neck lateral flexion in dominant shoulder side than non-dominant shoulder side when in a stationary position (d±90% CI = −0.64 ± 0.46)
		Neck rotation (°)	14 ± 10	16 ± 15	13 ± 11		—
		Trunk flexion (°)	52 ± 10	52 ± 11	52 ± 10		—
		Trunk lateral flexion (°)	23 ± 6	18 ± 5	20 ± 10		↑ trunk lateral flexion in dominant shoulder side in a stationary position than non-dominant shoulder side in a stationary position (d±90% CI = 0.92 ± 0.42) or dominant shoulder side when in-motion (d±90% CI = 0.33 ± 0.44)
		Trunk rotation (°)	23 ± 13	21 ± 15	21 ± 18		
	**Kinematic variable**		*Shoulder (normal) (n=21)*	*Low (n=22)*	*Shoulder and opposite-leg (n=13)*	*Low and opposite-Leg (n=10)*	
Kawasaki et al. [28]	Contact	Knee flexion (°)	72.1 (−78.1, −66.2)	79.2 (−86.7, −71.8)	52.4 (−56.4, −48.4)	59.4 (−65.7, −53.1)	
		Ankle extension (°)	31.4 (26.8, 36.0)	33.3 (28.4, 38.2)	15.9 (10.3, 21.5)	24.2 (15.4, 33.1)	
		Hip extension (°)	−76.3 (−83.4, −69.1)	−82.1 (−89.7, −74.5)	−79.2 (−92.3, −66.2)	−89.0 (−98.0, −79.9)	
		Hip abduction (°)	2.6 (−4.1, 9.2)	−0.4 (−6.0, 5.2)	−2.6 (−7.4, 2.3)	−6.6 (−9.9, −3.4)	
		Hip external rotation (°)	4.1 (−2.7, 10.8)	6.5 (−1.8, 14.8)	16.7 (6.2, 27.1)	12.2 (2.5, 21.9)	
		Trunk flexion (°)	53.4 (−57.8, −49.0)	58.3 (−63.1, −53.4)	55.9 (−64.2, −47.6)	46.8 (−53.4, −40.3)	
		Trunk lateral flexion (°)	−9.4 (−13.2, −5.5)	−3.0 (−7.6, 1.6)	−21.7 (−30.3, −13.1)	−21.8 (−30.7, −12.9)	↓ trunk lateral flexion to the side of the impact shoulder (normal) versus shoulder and opposite-leg tackle (*P*<0.01); and more prominent in low height tackle (*P*=0.01)
		Trunk rotation (°)	9.2 (5.0, 13.4)	6.0 (1.6, 10.5)	1.3 (-7.8, 10.5)	−7.3 (−10.2, −4.4)	↑ trunk rotation to the side of the impact shoulder during shoulder (normal) versus shoulder and opposite-leg tackle (*P*<0.01); and more prominent in low height (*P*=0.03)
		Trunk inclination (°)	3.9 (1.0, 6.8)	−8.4 (−11.9, -4.9)	3.3 (−0.1, 6.6)	−10 (−16.1, −3.9)	↓ trunk inclination in low than shoulder (normal) tackle (*P*<0.01)
		Neck extension (°)	34.6 (29.5, 39.8)	40.7 (35.2, 46.2)	29.0 (21.8, 36.2)	34.6 (25.2, 43.9)	
		Neck bending (°)	−8.0 (−11.1, −5.0)	−5.6 (−10.8, −0.4)	−8.2 (−14.6, −1.9)	−9.9 (−16.1, −3.7)	
		Neck rotation (°)	−5.9 (−9.9, −2.0)	-7.0 (-14.5, 0.5)	0.4 (−7.6, 8.5)	4.1 (−5.6, 13.7)	
		Shoulder external rotation (°)	59.3 (51.6, 66.9)	63.9 (57.1, 70.8)	56.7 (43.7, 69.6)	37.8 (21.9, 53.8)	
		Shoulder abduction (°)	59.3 (54.2 to 64.4)	66.2 (61.4 to 71.1)	58.2 (49.3 to 67.0)	42.2 (33.4 to 51.0)	
		Shoulder horizontal abduction (°)	−41.6 (−46.7, −36.6)	−41.2 (−49.2, −33.2)	−30.5 (−42.6, −18.3)	−29.1 (−43.8, −14.3)	
	Two steps before contact	Ankle extension (°)	0.6 (-3.1, 4.3)	0.6 (−4.9, 6.1)	3.7 (−4.7, 12.2)	19.6 (12.2, 27.1)	
		Knee flexion (°)	65.6 (−71.2, −59.9)	67.6 (−74.9, −60.4)	51.1 (−56.5, −45.7)	48.9 (−54.8, −43.0)	
		Hip external rotation (°)	1.2 (−3.6, 6.1)	1.4 (−6.6, 9.5)	0.9 (-5.2, 7.0)	11.9 (3.0, 20.8)	
		Hip abduction (°)	−5.3 (−7.6, −3.0)	−8.6 (−11.3, −5.9)	-5.9 (-9.3, -2.5)	−7.5 (−10.6, −4.4)	Hip abduction angles at the two steps before contact significantly influenced trunk inclination at the impact
		Hip extension (°)	−10.7 (−16.2, -5.3)	−13.5 (−19.8, −7.2)	−11.8 (−24.1, 0.5)	−90.6 (−98.2, −83.0)	
		Trunk inclination (°)	32.5 (28.2, 36.7)	24.8 (20.8, 28.8)	32.2 (26.5, 38.0)	8.2 (1.6, 14.8)	
		Trunk rotation (°)	0.4 (−3.7, 4.5)	−2.5 (−6.6, 1.6)	9.5 (5.9, 13.2)	−9.1 (−11.9, −6.4)	Trunk rotation contralateral to the side of the impacted shoulder at the two steps before contact significantly influenced trunk inclination at the impact
		Trunk bending (°)	−3.9 (−6.6, −1.2)	1.7 (−1.8, 5.1)	−5.9 (−14.2, 2.4	−3.5 (−17.9, −9.1)	
		Trunk flexion (°)	47.5 (-53.2, −41.9	58.1 (−64.0, −52.3)	48.2 (−54.0, −42.4)	52.6 (−58.7, −46.4)	
		Shoulder horizontal abduction (°)	−21.0 (−46.3, 4.2)	-19.8 (−44.4, 4.9)	−17.1 (−43.4, 9.3)	−33.2 (−46.2, −20.2)	
		Shoulder abduction (°)	32.0 (26.7, 37.3)	38.5 (33.9, 43.2)	32.4 (24.6, 40.2)	40.5 (30.6, 50.5)	
		Shoulder external rotation (°)	37.7 (28.9, 46.6)	43.5 (37.2, 49.8)	22.5 (10.3, 34.7)	32.5 (18.9, 46.1)	
		Neck rotation (°)	10.3 (8.2, 12.4)	10.2 (7.0, 13.4)	−2.5 (−6.8, 1.8)	8.2 (2.0, 14.4)	
		Neck bending (°)	4.6 (1.7, 7.5)	6.8 (3.3, 10.3)	−8.4 (−11.2, −5.5)	−12.3 (−17.6, −7.0)	
		Neck extension (°)	26.1 (17.7, 34.5	33.1 (27.7, 38.5)	28.0 (18.3, 37.7)	38.0 (29.2, 46.8)	
Tanabe et al. [27]	Contact		*Shoulder (n=32)*	*Arm (n=23)*	*Head-in-Front (n=4)*		
		Neck extension (°)	28 (22, 34)	28 (20, 36)	13.2 (6, 20) (sig)		
		Neck bending (°)	−12 (−16, −7)	−13 (−21, −4)	−5 (−20, 11)		
		Neck rotation (°)	−19 (−23, −15)	−18 (−26, −10)	−35 (−68, −3)		
		Shoulder external rotation (°)	54 (48, 60)	61 (49, 73)	33 (20, 47) (sig)		
		Shoulder abduction (°)	75 (68, 82)	95 (84, 107) (sig)	97 (59, 136)		
		Shoulder horizontal abduction (°)	−30 (−35, −26)	−36 (−43, −28)	−46 (−67, −25)		
		Trunk flexion (°)	59 (−63, −55)	59 (−66, −52)	55 (−65, −44)		
		Trunk bending (°)	−22 (−27, −17)	−13 (−23, −3)	−25 (−49, −1)		
		Trunk rotation (°)	4 (−2, 9)	10 (3, 16)	7 (2, 12)		

Note. *Sig* significant, *NS* non-significant, *NR* not reported

Reference [25] reported data as mean ± SD [28]; reported data as either mean (95% confidence interval) or odds ratio (95% confidence interval)

**Table 5:** Theme 5—findings of the studies: other three-dimensional variables of the tackler and/or ball carrier during a tackle task

	Event	Kinematics					Significant Findings
Kerr et al. [25]	After contact		*Skilled (male + female)*	*Novice (male + female)*	*Skilled (male + female)*	*Novice (male + female)*	
			*Pre-intervention*	*Pre-intervention*	*Post-intervention*	*Post-intervention*	
		Peak shoulder acceleration (m/s^2^)	482 ± 155	400 ± 103	381 ± 112	412 ± 114	Collegiate: NS
				Tackle 1	Tackle 2	Tackle 3	Collegiate: NS High school: linear head acceleration increased after video tackling instruction intervention (*P*=0.03) and decreased with repetition (*P*=0.01).
				*Pre-intervention*	*Pre-intervention*	*Pre-intervention*	
		Peak head linear acceleration (m/s^2^) (SEM)	Collegiate	293.2	—	—	
			High school players	75.1 (6.11)	62.7 (4.49)	60.3 (2.01)	
				Tackle 4	Tackle 5	Tackle 6	
				*Post-intervention*	*Post-intervention*	*Post-intervention*	
			Collegiate	—	—	—	
			High school players	70.0 (4.76)	64.0 (4.22)	64.7 (4.79)	
Tierney et al. [22]	After contact		*Upper-ball carrier*		*Low-ball carrier*		
		Linear head acceleration (m/s^2^)	78.9 ± 32.7 (30.7, 119.1)		57.5 ± 34.7 (28.4, 137.8)		
		Angular head acceleration (rad/s^2^)	354.1± 129.6 (179.3, 566.6)		203.7 ± 138.5 (64.03-394.9)		↑ angular head acceleration in Upper than Low (*P*=0.025, *d*=0.50)
		Change in head angular velocity (rad/s)	7.0 ± 2.4 (4.0, 10.9)		3.4 ± 2.2 (0.9, 7.3)		↑ change in head angular velocity in upper than low (*P*=0.004, *d*=0.64)
	Contact		*Upper*		*Low*		
		Tackler speed (m/s)	2.5 ± 0.6		2.1 ± 0.5		Moderate effect of tackler speed (*P*=0.125, *d*=0.72)
		Ball carrier speed (m/s)	1.7 ± 0.5		1.4 ± 0.6		Moderate effect of ball carrier speed (*P*=0.176, *d*=0.63)
Tierney and Simms [23]	After contact		*Mid/lower*		*Upper*		
		Ball carrier change in resultant head linear velocity (m/s)	0.98 (0.90, 1.12)		1.49 (1.25, 1.55)		
		Ball carrier change in head angular velocity (rad/s)	2.76 (1.79, 3.51)		6.80 (5.40, 8.63)		
Seminati et al. [26]	During tackle		*Dominant shoulder stationary*	*Non-dominant shoulder stationary*	*Dominant shoulder in-motion*		
		Total impact force (kN)	2.93 ± 0.74	2.57 ± 0.57	3.62 ± 0.79		↑ total impact force in dominant shoulder stationary than non-dominant stationary (d±90% CI = 0.53 ± 0.40) ↑ total impact force in dominant shoulder in-motion than dominant stationary (d±90%CI = −0.96 ± 0.44)
		Impulse of the total force (s)	0.102 ± 0.012	0.111 ± 0.021	0.095 ± 0.020		↓ impulse of total force in dominant shoulder stationary than non-dominant stationary (d±90%CI = 0.24 ± 0.42) ↓ impulse of total force in dominant shoulder in-motion than dominant stationary (d±90%CI = −0.27 ± 0.29)
		Contact time duration (s)	0.102 ± 0.012	0.111 ± 0.021	0.095 ± 0.020		↓ contact time in dominant shoulder stationary than non-dominant stationary (d±90%CI = −0.56 ± 0.36) ↓ contact time in dominant shoulder in-motion than dominant stationary (d±90%CI = 0.47 ± 0.42)
Kawasaki et al. [28]	Contact		*Shoulder*	*Low*	*Shoulder and opposite leg*	*Low and opposite leg*	
		Step distance (%)	20.7 (14.5, 26.9)	17.2 (10.7, 23.7)	15.6 (6.4, 24.7)	25.6 (20.3, 30.9)	
		Shoulder height (%)	43.7 (42.4, 45.1)	34.6 (33.1, 36.0)	43.2 (45.2, 45.3	33.7 (32.3, 35.1)	
Tanabe et al. [27]	Before contact		*Shoulder (n=23)*	*Arm (n=38)*	*Head-in-front (n=4)*	Total (*n*=65)	
		Run straight *n* (row%)	32 (84.2)	10 (43.5)	4 (100)	46 (70.8)	↑ arm tackle frequency significantly if the ball carrier changed his course compared to shoulder tackle (odds ratio, 6.9; *P*<0.001)
		Cutting (row%)	6 (15.8)	13 (56.5)	0 (0)	19 (29.2)	
Wundersitz et al. [24]	Contact		*Tackle bag (n=250)*	*Bump pad (n=250)*	*Tackle drill (n=125)*		
		Peak impact acceleration of inertial measurement unit (G) mean, SD	7.24 ± 1.65	4.79 ± 1.58	6.00 ± 1.93		

Reference [22] reported data as medians and upper and lower quartiles [23]; reported as absolute median (25% and 95% confidence interval) [25]; reported data as mean ± SD/SEM or as a mean and an effect size [26]; reported data as mean ± SD [27]; reported data as mean (95% confidence interval) or as *r* [28]; reported data as either mean (95% confidence interval) or odds ratio (95% confidence interval) or as a mean

### Video Tackling Instruction Intervention

Kerr and colleagues [25] observed a significant 40° increase in the tackler’s knee joint angle at impact pre- compared to post-video tackling instruction intervention in skilled male and female college players (*n*=9), yet this was not observed in novice male or female players (*n*=5). Peak shoulder and cervical spine linear accelerations were not altered following a video tackle technique instruction in collegiate players, but cervical spine linear acceleration increased post-intervention in high school players [25].

### Shoulder Side Engagement

At impact, when community and university players tackled a bag from a stationary position and engaged with their dominant shoulder, their neck flexion decreased by 5°, neck lateral flexion decreased by 6°, and trunk lateral flexion increased by 5° compared to their non-dominant shoulder engagement [26]. When these players used their dominant shoulder to engage in the tackle from a stationary position compared to an in-motion start, they displayed 5° less neck flexion and 3° less trunk lateral flexion [26].

### Ball Carrier Head Acceleration Changes with Torso Contact Height

The ball carrier displayed a significantly higher head angular acceleration and change in angular velocity but not in linear head acceleration when contact by the tackler was made to their upper trunk, compared to mid/lower trunk tackle [22]. The change in the ball carrier’s resultant head linear and angular velocity during the tackle was individually reported for nine mid/lower body tackles, and 11 upper torso tackles were reported [23].

### Upper Body Kinematics Change with Tackle Torso Height and Leading Lower Limb

College and professional players’ forward trunk inclination angle at impact with a tackle bag was significantly influenced by the hip abduction angle of the leading leg and trunk rotation away from the contacted shoulder two steps prior to impact [28]. When these players performed this task at a low tackle height, compared to normal tackle height, they increased their forward trunk inclination angle by approximately 15°, irrespectively of whether they used their left or right leg as their leading leg at impact. The players in a low tackle also increased their trunk rotation and decreased their lateral trunk flexion to the side of impact compared to a normal tackle height [28]. Whether at impact the leading leg was the same side (i.e. right shoulder, right leg) or opposite side (i.e. right shoulder, left leg) that was used to execute the tackle significantly affected the magnitude of trunk lateral trunk flexion and rotation to the side of impact [28]. The tackler’s shoulder height and step distance when tackling a tackle bag were not significantly different for any of the four tackle conditions examined [28].

### Type of Tackle (Arm, Shoulder, Head-in Front)

The frequency of a change in the approach movement of the ball carrier to a ‘cut action’ was significantly greater in the arm tackle condition than in the shoulder tackle condition (i.e. the reference condition) [27]. An arm tackle displayed larger shoulder abduction angle but no difference in any other neck, trunk, or shoulder angles at contact when compared to a shoulder tackle [27]. When a player performed a head-in-front shoulder tackle rather than a traditional shoulder tackle in this study, the tackler decreased their amount of neck flexion and shoulder external rotation [27].

### Player Speed

The impact speed of the ball carrier was found to be higher, though not significantly, when the tackler made contact with the ball carrier’s upper trunk, as opposed to the ball carrier’s mid/lower trunk [22]. A significantly lower mean impact force of a tackler when tackling a tackle bag was shown in the stationary start condition in the non-dominant shoulder versus a dominant shoulder, and in the dominant shoulder in the stationary start condition versus the in-motion start condition [26]. The contact time duration was significantly reduced during tackling in the ‘in-motion start, dominant shoulder’ tackle condition compared to the ‘stationary start, dominant shoulder’ tackle condition, as well as for the ‘stationary start, dominant shoulder’ tackle condition than the ‘stationary start, non-dominant shoulder’ [26].

### Validation of Triaxial Accelerometer

One study was focused on methodology to identify the most appropriate data reduction method when processing peak acceleration recorded from a triaxial accelerometer device unit during a tackle [24].

## Discussion

**Fig. 2:**
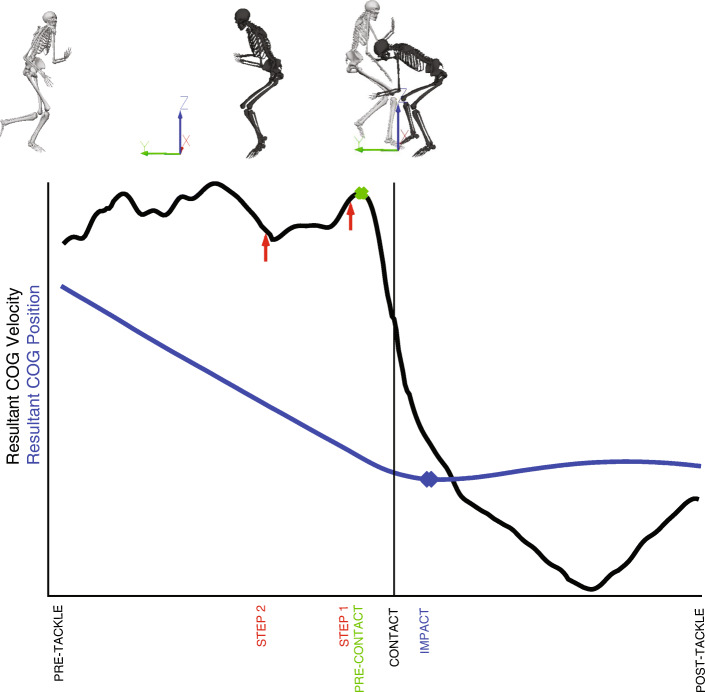
Recommendations for undertaking 3D motion analysis of the tackle technique

This is the first review to integrate 3D motion capture rugby-style tackle technique research. Seven studies met the eligibility criteria for inclusion in this review. Eligible studies were of moderate (*n*=1) or high (*n*=3) risk of bias, due largely to small participant numbers, study design, outcome limitations and the inclusion of questions within the bias of risk assessment not relevant to 3D motion studies that skewed the risk of bias score. Quantitative analysis was not feasible due to the heterogeneity of study design and sample cohorts across these studies, and led to our pre-specified qualitative synthesis using thematic analysis. Key themes identified within this review enable the development of the subsequently described research framework for 3D tackling biomechanics research. *Research framework recommendations* are to inform methodological practice, advancing biomechanical knowledge of injury risk and performance during the multidirectional dynamic game-play movement of tackling, and are outlined in Fig. 2. This research framework has four key research recommendations: (i) participant demographic information, (ii) experimental task design information, (iii) 3D motion capture data specifications, and (iv) 3D data reduction and analysis. These four areas must be adequately reported to compare results across studies and ensure replication of study design is possible.

### Theme One—Participant Demographic Information

All eligible studies included male rugby union players as participants. One of these studies [25] also included female participants. Considering the emergence of female participation in the rugby codes [29], further epidemiological research is required to determine if, and the extent to which, limited technical tackling training and/or experience [29, 30] may contribute to any disparity in injury rates between male and female players.

Coaching manuals present different tackle techniques between the rugby codes [31, 32]. Within rugby codes, there are also age-related tackle technique differences [33], in addition to a range in skill level [33, 34], playing experience [34] and various demand contingent upon playing position [35]. A major shortcoming of this body of work is the failure to report comprehensive information pertaining to participant demographics. One underpowered study investigated the effectiveness of a coaching intervention (i.e. watching a tackling instruction video) for a rugby player’s one-on-one, front-on tackling technique [25]. The aim of this study was to determine whether watching an education video would alter a tackler’s knee and hip angles at contact. The authors undertook inappropriate statistical analysis with their limited sample size using variables displaying large standard deviations, such as comparing male novice versus skilled player’s knee flexion angle at impact (novice *n*=4, 65.5 ± 5.1°; skilled *n*=3, 103.2 ± 20.1°; *p*=0.014). Given the study design and methodology limitations, the absence of essential experimental task descriptions (tackle speed, tackle intensity), 3D motion collection and analysis (sampling frequency, 3D model), it was not possible to verify that their coaching intervention altered tackle technique.

### Theme Two—Experimental Task Design Information

Eligible studies investigated either one-on-one, front-on [22, 24, 25, 27] or side-on [27] contact between a tackler and a ball carrier or a tackler impacting a tackle bag or bump pad [24, 26, 28]. The tackle event can be confounded by numerous factors including the number of tacklers performing the tackle, whether the tackle is taken to the ground, the intensity of the tackle and player speed. One limitation of focusing solely on the one-on-one tackle is that the results cannot be extrapolated to multi-player tackles, which is the most common tackling situation in rugby league [10] and rugby union [36] match play. Whether the tackle is taken to the ground [24, 25] (41% of tackles in rugby union [33]) or not [26] is often unreported [22, 23, 27, 28]. Measuring 3D tackle motion of multi-player tackles and tackles taken to the ground is a feasibility issue with optoelectronic 3D motion capture system due to marker occlusion and concerns about the skin-mounted retroreflective markers.

Although most match play injuries occur at a ‘slow tackle speed’ (64%) [36], there is a higher relative risk of injury [36], concussion [37] and other head impact injury [38] with a higher tackle speed at contact. Despite this, only one of the seven eligible studies reported the impact speed of the tackler and ball carrier at contact when the tackler used a 2.5-m approach distance (but unknown tackle intensity instruction), when performing a one-on-one, front-on tackle [22]. This tackle speed would be categorised as slow [36]. It is well-known that speed alters kinematics and kinetics in other movements such as gait [39] and a change-of-direction task [40]. The influence of speed on tackle technique was highlighted in a study using a tackle bag with the tackler in a motion rather than in a stationary position. This study reported that the player increased their total impact force, decreased their contact time, increased their neck flexion and decreased their trunk lateral flexion when in motion compared to the stationary start position [26]. Speed of the tackler can be influenced by the experimental task instructions such as tackle intensity, approach distance (greater distance, higher speed), number of tacklers involved in the tackle, the surface and the actions of the ball carrier and other opponents in the tackle. These variables are commonly absent from the methodological description of studies. It is important that researchers clearly articulate their methodology on the factors that can alter the tackler’s speed to improve the interpretation of study results.

### Theme Three—3D Data Collection

The quality of optoelectronic 3D motion capture data is influenced by the individual laboratory setup [41, 42]. Critical factors affecting accuracy such as the number of cameras [41] and marker set [43] were universally reported by studies included in this review. Marker sets need to be able to measure key 3D outcome variables in tackle research. For example, when tackling, a neutral spine or ‘straight back’ posture is recommended [31] for performance [44] and to reduce [45] head and spinal [46] injury risk. This 3D spinal alignment could not be quantified in five studies [22, 23, 26–28] with their custom [26–28] or plug-in gait [22, 23] marker set. These marker sets modelled the thorax as a single segment [47] rather than as a two segment (lumbar and thoracic) spine model that can measure thoracolumbar and lumbopelvic joint angles [48] to quantify a straight back posture. Only head segment [22, 23, 26–28] and not neck segment [49] could be modelled with these marker sets, despite one study incorrectly labelling their neck joint angle that was actually the head segment relative to the trunk segment angle [26]. Cervical angle reported by one study [25] was a three-point 2D planar angle that cannot measure the 3D spinal motion.

**Fig. 3:**
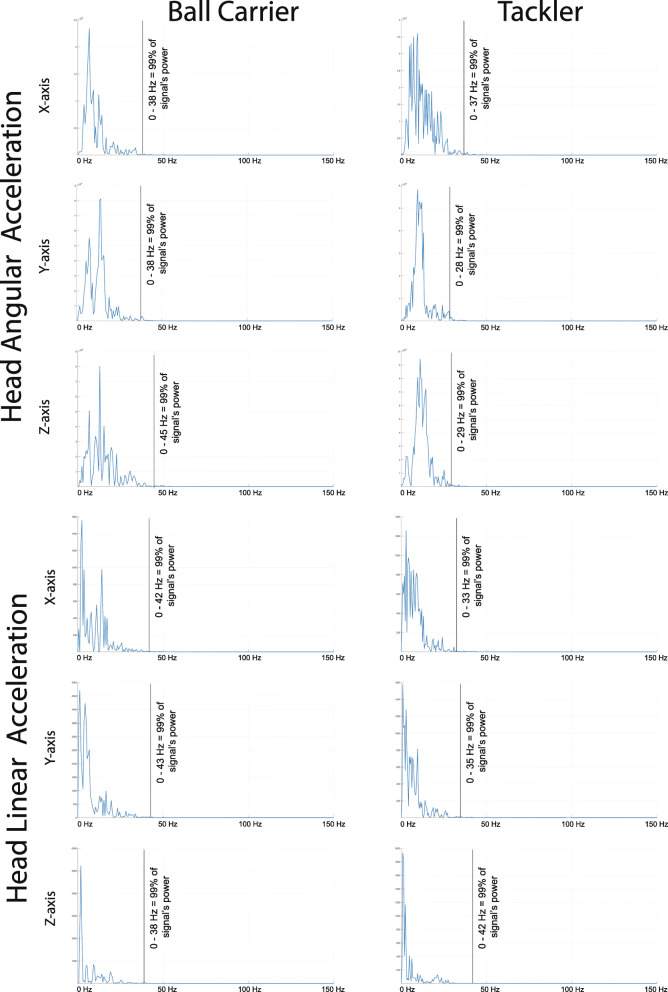
An example of spectral analysis of a ball carrier and tackler within a single trial of a front-on, one-on-one tackle (unpublished data)

The data collection volume [42] was reported in three of the studies [25, 27, 28], and one study did not report sampling frequency [25]. The paramount importance of this data collection information is exemplified in the parameter sampling frequency. When a researcher is selecting the sampling frequency, they must adhere to the Nyquist sampling theorem to avoid aliasing errors in which the data (i.e. 3D position of the markers) must be set as twice the highest frequency within the data [50]. Considering the importance of measuring head acceleration during a tackle to understand head impacts, if you set a sampling frequency too low, you will miss measuring the true peak head acceleration magnitude due to the aliasing error and thus make the results redundant. Spectral analysis [50] of 3D head linear and angular acceleration during front-on, one-on-one torso tackle at a slow tackle speed (unpublished) indicates that the median frequency in which 99% of the data occurs below is 49 Hz (Fig. 3). Thus, a sampling rate ≥ 200 Hz is recommended when the primary outcome variable is head acceleration. These data collection parameters were met for all but one study [25].

Passive optoelectronic motion capture systems were employed by all studies within this systematic review. Other types of 3D motion capture system technology to measure human motion in sport exist, but no 3D motion capture system currently exists that can measure large volumes with high accuracy [42] within a game environment. Technological advances in 3D motion capture technology offer the potential for in-game rugby-style tackle technique to be assessed in future research. Model-based image matching is one such emerging technology that uses multiple 2D video camera views and applies a multibody skeletal model to estimate in-game 3D player kinematics [51] or 3D head velocity during a tackle in rugby union [52] and American football [53]. This manual process also takes 60 h to complete a single case study [54]. Markerless image processing technology to estimate 3D joint angles is valid [51] but not 3D head velocity [54]. It is recommended that due to the low sampling rate of the broadcast video of 100 Hz [54] or game video footage of 25 Hz in rugby union [52] or 60 or 120 Hz in American football [53], neither head velocity nor acceleration should be calculated from broadcast video as its low sampling rate violates Nyquist sampling theorem based on spectral analysis. An alternative technology measuring head acceleration is wearable sensors such as instrument mouthguards [55]. Nevertheless, further technological development of this markerless motion capture system technology using human pose estimation and wearable sensors offers a real-world in-game solution to quantify in-game 3D tackle mechanics and should be further explored.

### Theme Four—3D Data Reduction and Analysis

Variations in the data reduction and analysis methodology can alter results. For example, using different methods of joint modelling [56], filtering [57] and definition of kinematic variables [58] have been shown to produce varying results. Poor quality reporting of methodological detail complicates the interpretation of study results. For example, Kerr and colleagues [25] did not report their data sampling frequency, the filtering methods of their 3D motion capture data, and utilised a marker suit rather than attaching the markers to the participant’s skin. These methodological shortcomings render interpretation of the accuracy of the kinematic data impossible to ascertain. Specifically, it is not known whether (i) the Nyquist sampling theorem was violated, (ii) the lack of filtering introduced extraneous noise in the data, (iii) the relative movement between the marker suit and skin decreased the reliability of the data, (iv) the magnitude of the error when modelling the 3D body segments and joint centres when using suit markers that roughly estimate the underlying anatomical landmark in participants of differing anthropometric dimensions, (v) the study used the joint coordinate system of Grood and Suntay [59] or the International Society of Biomechanics [60], and/or (vi) the Cardan sequence of rotation to express inter-segment joint angles was used and the sequence of rotation selected if used.

A zero-lag, fourth order Butterworth low pass filter was commonly employed (six studies), yet no justification for cutoff frequency selection was provided in any of those studies. The appropriate cutoff frequency can be identified via residual analysis of the raw kinematic data to attain a balance between signal distortion and noise within the data [50]. Residual analysis [50] of raw 3D position of head markers during a front-on, one-on-one torso tackle at a slow tackle speed (unpublished) showed a median frequency of 12 Hz (range 9 to 14 Hz); recommending a cutoff frequency ≥15 Hz should be used when using a zero-lag, fourth order Butterworth low pass filter. Though some eligible studies used a suitable 15 [26] or 16 Hz [22, 23] cutoff frequency, two studies oversmoothed their data with a selection of 6 Hz cutoff frequency [27, 28], a process that causes problems with signal distortion.

The description of the algorithm employed to identify temporal events is another important methodological detail that is often not sufficiently reported to enable replication of methods. For example, Kawasaki and colleagues [27, 28] used ‘initial displacement of the markers’ as the criteria for the time of impact; however, it was not specified which markers were used, nor the threshold value used to define ‘initial displacement’ (i.e. how far the markers were required to move to define the temporal event). Work by Schaefer and colleagues [61] in cricket fast bowling (see their Additional file 1: Appendix 1 to 3) illustrates the ideal level of detail of data reduction and analysis methodology required for 3D kinematics research (i.e. marker set information, definitions of each segments mass and inertial properties, how the markers set are used to define the segments, specifications of the joint coordinate systems and 3D rotations [e.g. cardan sequence], and defining temporal events).

### Theme Five—Findings of Studies

Several between-study methodological differences made it challenging and inappropriate to undertake comparisons of findings in this review. Some of the key findings across these studies are summarised below.

Four studies examined how modification of the tackle technique alters a player’s mechanics during a tackle. Two studies investigated tackle height, which was categorised as either a high or low tackle [22, 28]. Tackle height is a critical aspect of the tackle to investigate. The majority of head impact injuries occur during a high tackle as opposed to a low tackle for both the tackler and ball carrier [38, 62], with the tackler sustaining most of the head impact injuries rather than the ball carrier [37, 38]. Kawasaki and colleagues [28] observed that at impact when players contacted a tackle bag at a low tackle height, they increased their angle of trunk inclination and decreased their trunk lateral flexion and trunk rotation angle to the side of the impacted shoulder compared to a high tackle height. The authors also reported that the player’s trunk inclination at impact was significantly influenced by the magnitude of hip adduction and trunk rotation contralateral to the side of the impact two steps prior to impact. Tierney and colleagues [22] found that when a ball carrier was tackled in a one-on-one front-on tackle, they experienced higher head angular acceleration and change in angular velocity in high compared to a low tackle.

A tackler maintaining ‘leg drive upon contact’ has been associated with a decreased risk of a sustaining a concussion [63, 64] and an increased capacity to reduce the progression of the ball carrier [44]. To improve the clarity of the role of the leg drive within the tackle, Kawasaki and colleagues [28] investigated whether using a different leading leg when contacting a tackle bag altered the trunk mechanics of the tackler. When players used the same side leading leg during the tackle (i.e. right shoulder, right leg), players were observed to decrease their amount of trunk lateral flexion and increase their trunk rotation to the side of the impact shoulder compared to when using the opposite leading leg. The only other study investigating variables associated with leg drive mechanics reported descriptive data of knee and hip joint kinematics [25]. Knee flexion, but not hip flexion, was found to be altered in skilled, but not novice players, after watching a tackling instruction video [25].

Three studies attempted to understand whether upper limb injury risk was altered when engaging with dominant or non-dominant shoulder when tackling a bag [26] or differed with the type of one-on-one tackle [27]. Seminati and colleagues [26] reported that players engaging in dominant shoulder contact with a tackle bag had decreased neck flexion and lateral neck flexion, and increased trunk lateral flexion, compared to non-dominant shoulder engagement. This was found to result in decreased total contact force and longer contact duration. Arm tackles are attributed to the most number of shoulder injuries when tackling [36] and occur more frequently when the ball carrier changes their running direction [27]. The results of research by Tanabe and colleagues [27] reported a larger shoulder abduction angle, yet no difference in any other neck, trunk or shoulder angles at contact when compared to a shoulder tackle. When a player performed a head-in-front shoulder tackle rather than a traditional shoulder tackle in this study, the tackler decreased their amount of neck flexion and shoulder external rotation.

One of the limitations of the current review was that limiting eligibility for inclusion to only peer-reviewed journal articles excluded some recent conference proceedings such as [65].

## Conclusion

An urgent need for high-quality 3D motion capture studies investigating 3D tackling mechanics in the rugby codes is warranted, ideally using a research framework proposed in this review. The lack of adherence by the present body of research to all key parameters of this research framework (participant demographic information, experimental task design information as well as 3D motion capture data collection, and reduction and analysis methodologies) confound these studies’ findings. Only limited laboratory-based evidence using passive optoelectronic 3D motion capture currently exists on the 3D biomechanics of tackling techniques to guide coaches and clinicians on the most optimal method to execute a tackle to reduce the injury risk and optimise performance for both the ball carrier and tackler. Overcoming the current methodological challenges in measuring accurate in-game 3D tackle mechanics may be overcome in the future by technological advances in 3D motion capture technology that should be further explored, such as markerless image processing using human pose estimation and wearable sensors.

## Supplementary Information

**Additional file 1.** Appendix.

## Data Availability

All materials are listed within this manuscript.

## Abbreviations

2D: Two-dimensional
3D: Three-dimensional motion
NRL: National Rugby League
CI: Confidence interval
PRISMA: Preferred Reporting Items for Systematic Reviews and Meta-Analyses (PRISMA)
