# Supplementary material for: 3D Biomechanics of Rugby Tackle Techniques to Inform Future Rugby Research Practice: a Systematic Review
Source: Sports Med Open. 2021 Jun 7;7:39. doi: 10.1186/s40798-021-00322-w (PMC8184906; doi:10.1186/s40798-021-00322-w)

**Appendix 1**

**Search Strategy and Results Overview**

| **Database** | **# results** | **Search String used** | **Comments** |
| --- | --- | --- | --- |
| Medline | 130 | See screenshot below |  |
| SPORTDiscus | 157 | ( biomechanic* OR 3D OR three-dimensional OR video OR kinematic* OR kinetic* ) AND ( tackl* OR impact* OR collision* ) AND rugby | Searched ‘all fields’ except full-text  Limited to Peer Reviewed and English Language |
| Web of Science | 204 | ( biomechanic* OR 3D OR three-dimensional OR video OR kinematic* OR kinetic* ) AND ( tackl* OR impact* OR collision* ) AND rugby | Searched ‘Topic’ which searches title/abs/keywords  Limited to English Language |
| Scopus | 245 | ( biomechanic* OR 3D OR three-dimensional OR video OR kinematic* OR kinetic* ) AND ( tackl* OR impact* OR collision* ) AND rugby | Searched ‘title-abstract-keyword’  Limited to English Language |
| CINAHL (EBSCO) | 113 | ( biomechanic* OR 3D OR three-dimensional OR video OR kinematic* OR kinetic* ) AND ( tackl* OR impact* OR collision* ) AND rugby | Searched across all fields |
| ProQuest Research Library | 69 | ( biomechanic* OR 3D OR three-dimensional OR video OR kinematic* OR kinetic* ) AND ( tackl* OR impact* OR collision* ) AND rugby | Searched all fields except full text.  Limited to Peer Reviewed and English Language |
| PubMed | 390 | ( biomechanic* OR 3D OR three-dimensional OR video OR kinematic* OR kinetic* ) AND ( tackl* OR impact* OR collision* ) AND rugby | Searched all fields except full text.  Limited to Peer Reviewed and English Language |
| Embase | 87 | See screenshot below | Limited to English Language |
| Cochrane Library | 7 | ( biomechanic* OR 3D OR three-dimensional OR video OR kinematic* OR kinetic* ) AND ( tackl* OR impact* OR collision* ) AND rugby |  |
| **Total** | **1498** |  | **All searches completed 13.5.2020** |

**Medline results 13.5.2020 – 143 results**


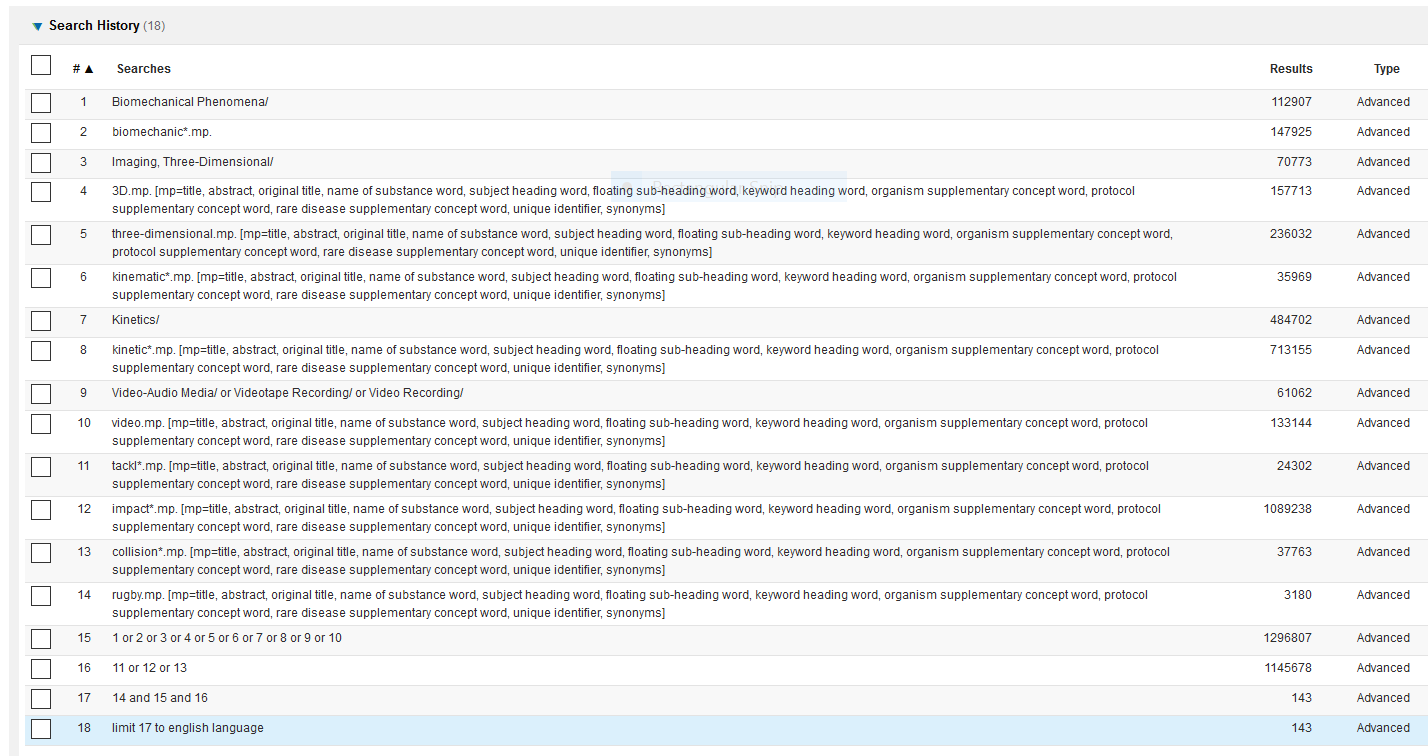


**EMBASE Search 13/5/2020– 170 results**


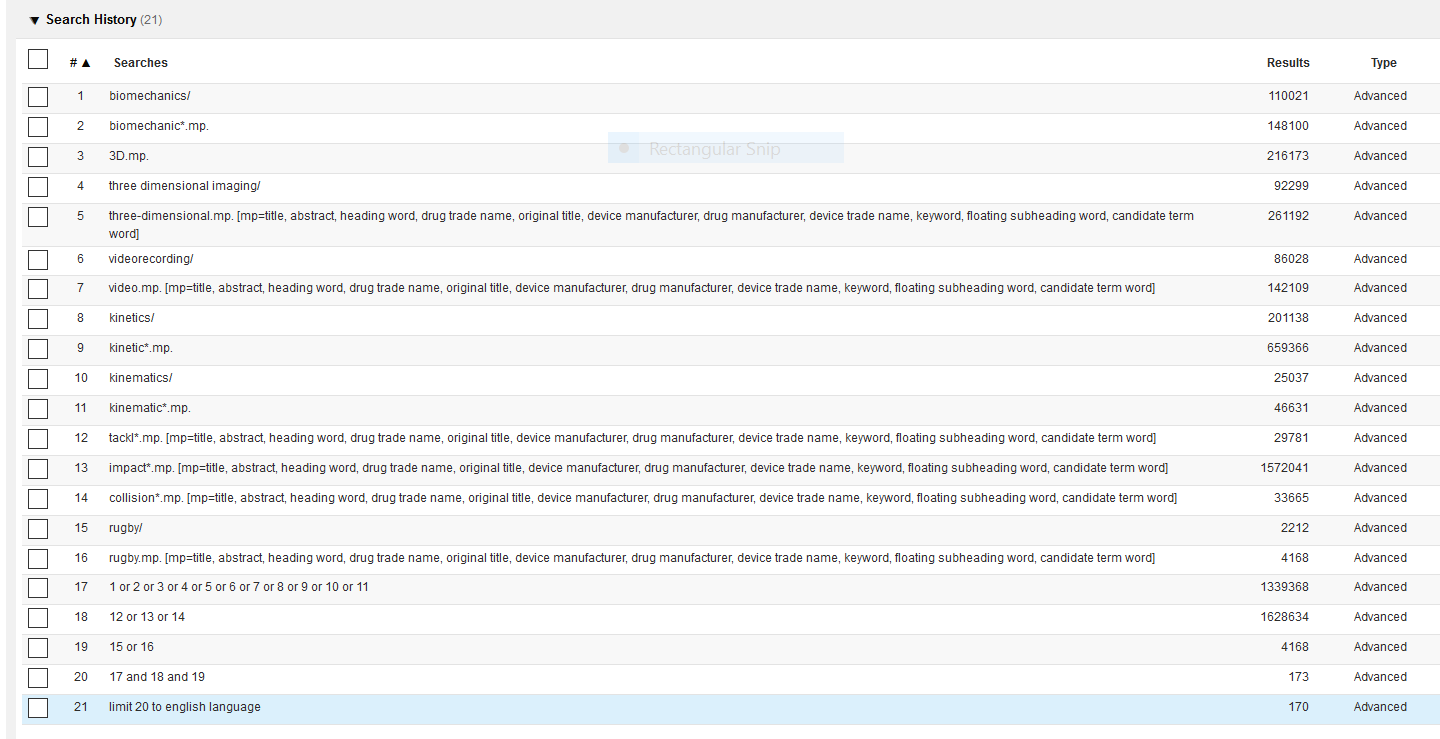

Supplement: Supplementary file 1 — Additional file 1. Appendix. [file 40798_2021_322_MOESM1_ESM.docx]
